# Training the Professor: Health Policy Curriculum in Adult Development and Aging Course

**DOI:** 10.1093/geroni/igaf122.3509

**Published:** 2025-12-31

**Authors:** Kirsten Graham, Sam Webster, Kelly O’Malley

**Affiliations:** Southern Utah University, Cedar City, Utah, United States; Southern Utah University, Cedar City, Utah, United States; VA Boston Healthcare System, Boston, Massachusetts, United States

## Abstract

Health policy shapes the lives of older adults but is often underrepresented in undergraduate psychology curricula. This study builds on our prior implementation research integrating health policy content into an undergraduate psychology course. Previous research (student reflections, instructor observations, and course assessments) showed significant increases in students’ knowledge, understanding of health policy-making, and critical evaluation skills. Building on these results, we developed a Train the Professor package to help faculty incorporate policy content into similar courses. This package includes a structured curriculum planning framework, consultation with a psychologist experienced in policy work, and ready-to-use instructional materials. We also evaluated the Knowledge and Attitude about Social Policy (KASP) scale, which was developed and revised as part of the curriculum evaluation. Confirmatory factor analyses pre (𝜒2 (8) = 12.0 p = .152; CFI = 0.993; TLI = 0.988; RMSEA = 0.051; SRMR = 0.031) and post (𝜒2 (8) = 12.0 p = .151; CFI = 0.992; TLI = 0.985; RMSEA = 0.057; SRMR = 0.033) indicated that the KASP was a good fit for the data. Findings support integrating policy education into psychology curricula and refining a scale for future research. By equipping educators with these resources, we aim to foster interdisciplinary learning and better prepare students to engage with policy issues related to aging. Our findings contribute to curriculum development discussions in psychology, emphasizing the value of policy integration in enriching student learning and better serving the community.

